# Protective effects of an aqueous extract of *Protaetia brevitarsis seulensis* larvae against radiation‐induced testicular injury in mice

**DOI:** 10.1002/fsn3.2992

**Published:** 2022-09-20

**Authors:** Hyeon‐Hwa Nam, Sohi Kang, Yun‐Soo Seo, Jun Lee, Byeong Cheol Moon, Hae June Lee, Ji Hye Lee, Bohye Kim, Sueun Lee, Joong‐Sun Kim

**Affiliations:** ^1^ Herbal Medicine Resources Research Center Korea Institute of Oriental Medicine Naju Korea; ^2^ College of Veterinary Medicine and BK21 Plus Project Team Chonnam National University Gwangju Korea; ^3^ Divison of Radiation Biomedical Research Korea Institute of Radiological and Medicinal Sciences Seoul Korea; ^4^ College of Korean Medicine Semyung University Jecheon Korea

**Keywords:** oxidative stress, protaetia brevitarsis seulensis, radiation, sperm, testis

## Abstract

The larvae of *Protaetia brevitarsis seulensis* have been used as a food ingredient and are known for their nutritional value and anti‐inflammatory properties. However, whether *P. brevitarsis seulensis* larvae demonstrate protective effects against radiation‐induced testicular injury has not been investigated. In this study, the protective effects of an aqueous extract of *P. brevitarsis seulensis* larvae (PBE) against radiation‐induced testicular injury were tested. Male C57BL/6 mice were administered PBE (5 or 10 mg/kg) orally for 14 days before exposure to focal pelvic irradiation. Histopathological examinations were conducted at 8 h and 30 d after radiation exposure. PBE pretreatment reduced the radiation‐induced apoptosis of germ cells at 8 h after irradiation and significantly increased testis and epididymis weights relative to those of the irradiated control mice at 30 days. PBE protected against histopathological damage and decreased the radiation‐induced effects on the epithelium height and seminiferous tubule diameter. Furthermore, the extract ameliorated the radiation‐induced morphological abnormalities of sperm cells and improved their motility. It also prevented a decrease in the epididymal sperm count caused by irradiation. Moreover, the extract alleviated the generation of reactive oxygen species, and its antioxidative activity increased in a dose‐dependent manner. Among the six major compounds isolated from PBE, benzoic acid and uridine showed the highest antioxidant activities. These results suggest that PBE protects against radiation‐induced testicular injury via its antioxidative properties. Thus, it has potential clinical applicability as a neoadjuvant therapy for the prevention of testicular damage caused by cancer radiotherapy.

## Graphical abstract

Our results showed that pretreatment with PBE inhibited detrimental testicular injuries, improved sperm quantity and quality, and significantly increased the number of seminiferous tubules. Benzoic acid and uridine were the primary constituents of PBE which showed the highest antioxidant activities. Therefore, PBE has potential applicability in providing protection against testicular injury from radiotherapy.

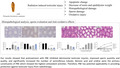

## 1 INTRODUCTION

The survival rates of patients with cancer have risen as a result of the progress made in radiotherapy, which remains a key method for the treatment of many malignant tumors. However, radiotherapy is associated with various side effects, including sore skin, fatigue, hair loss, vomiting, diarrhea, depression, and infertility (Hofman et al., 2004; Khan & Alhomida, 2011). In particular, for fertile male patients with Hodgkin's lymphoma, leukemia, and cancers of the reproductive organs (prostate, penile, and testicular cancers), radiotherapy can result in short‐term, long‐term, or even permanent damage to the sexual glands, resulting in germ cell loss, Leydig cell dysfunction, and even the complete cessation of spermatogenesis in severe cases (Rozati et al., 2017; Trost & Brannigan, 2012). Damage to the sexual glands can occur through direct exposure of the testes to radiation or, more commonly, scattered radiation during treatment targeted at adjacent tissues, such as the prostate and penis, with the degree of damage depending on the radiation dosage and delivery method (Okada & Fujisawa, 2019; Trost & Brannigan, 2012). The recommendation for spermatogenesis after radiotherapy is unpredictable. Studies of spermatogenesis in long‐term cancer survivors have shown that permanent azoospermia or serious oligozoospermia occurs in up to 24% of the patients (Andreu et al., 2000). Sperm cryopreservation prior to cancer therapy has been made clinically available in preparation for the eventuality of infertility in male cancer patients. Although this technique is well established, effective, inexpensive, and noninvasive, its application is limited to prepubescent boys, and ethical controversies over sperm banking still exist (Rozati et al., 2017). Therefore, supplementary methods, which can minimize radiation‐induced testicular damage in vulnerable male patients, are required.

Radiation can kill tumor cells and the surrounding healthy tissues by damaging DNA directly through ionization or indirectly via reactive oxygen species (ROS) generation (Kim et al., 2019). In particular, radiation with low linear energy transfer, such as X‐rays and gamma rays, can damage biological macromolecules indirectly, following the generation of ROS (Panganiban et al., 2013). These free radicals, especially superoxide anions (O_2_^−^), hydroxide radicals (OH^−^), and hydrogen peroxide (H_2_O_2_), produced by the radiolysis of intracellular H_2_O, can oxidize biological macromolecules (including DNA, protein, and lipids) and impair subcellular organelles (such as the endoplasmic reticulum and mitochondria) (Chatterjee et al., 2018; Srinivas et al., 2019). Therefore, oxidative stress caused by radiation constitutes a risk factor for inducing testicular damages. However, naturally occurring endogenous antioxidative systems (i.e., enzymatic and nonenzymatic antioxidants) protect against radiation‐induced oxidative damage by scavenging free radicals. Several studies have been reported on the association between antioxidant activity and testicular damages (Moghimian et al., 2017; Majid Shokoohi et al., 2022; Shokoohi et al., 2020; Soltani et al., 2018).

Insects constitute the largest group of organisms on Earth, and humans have been using insect bodies, larvae, eggs, eggshells, and secretions as chemical materials, foods, and medicines for a long time (Feng et al., 2009). For example, the larvae of *Protaetia brevitarsis seulensis* (Coleoptera: Scarabaeidae: Cetoniinae), commonly known as the white‐spotted flower chafer, have been used in traditional medicine across China, Taiwan, Japan, and Korea to treat various diseases, such as hepatitis, liver cirrhosis, breast cancer, tetanus, thrush, and toxic epilepsy (Lee et al., 2017; Yeo et al., 2013). Furthermore, *P. brevitarsis seulensis* larvae is a high protein resource containing fat (15%–17%) and protein (44%–58%), and the larvae of this insect species have recently been registered as a food ingredient by the Ministry of Food and Drug Safety of Korea (MFDS), owing to its nutritional value and biological activities, such as its anti‐inflammatory properties (MFDS, 2021; Baek et al., 2021). However, whether *P. brevitarsis seulensis* larvae have protective effects against radiation‐induced testicular injury has not been investigated.to date. As the antioxidant effects of *P. brevitarsis seulensis* ‐derived compounds and proteins were reported in various experimental models, an improvement of testicular dysfunction through the antioxidant properties of larvae‐derived products was expected (Ganguly et al., 2020; Lee et al., 2021). Therefore, in this study, we used a mouse model of radiation‐induced testicular injury to investigate the ameliorative effect of an aqueous extract of *P. brevitarsis seulensis* larvae (hereinafter PBE) on testicular dysfunction, and to identify which component of the extract is involved in this mechanism.

## 2 MATERIALS AND METHODS

### 2.1 Preparation of the aqueous larval extract

Dried larvae of *P. brevitarsis seulensis* were obtained from Kwang Myong Dang Co. (Ulsan, Korea) and were morphologically and genetically identified as described in a previous study (Lee et al., 2021). The samples (Manufacturer's No.: K2281201707) were deposited in the Korean Herbarium of Standard Herbal Resources (Index Herbariorum code: KIOM) at the Korea Institute of Oriental Medicine, Naju, Korea (Medicinal ID: 2‐18‐0111).

PBE was prepared using the methods described in a previous study (Lee et al., 2021). In brief, 887.4 g of dried *P. brevitarsis seulensis* larvae were ground; the grains were then extracted with 15 L of distilled water under reflux at 100 ± 2°C for 3 h. Thereafter, the extract was filtered, vacuum evaporated, and freeze‐dried to obtain the crude form of the extract (242.8 g, 27.4%), which was then stored at −20°C for this study.

### 2.2 Experimental animals and treatment schedules

Male C57BL/6 mice (7–8 weeks old and 23–25 g) were obtained from DooYeol Biotech (Seoul, Korea). All mice were housed in a room maintained at a temperature of 23 ± 2°C and relative humidity of 50 ± 10%, with artificial lighting from 08:00 to 20:00 and 13–18 air changes per hour. Mice were fed a standard animal diet. All animal experiments were carried out using a protocol approved by the Institutional Animal Care and Use Committee of the Korea Institute of Oriental Medicine (KIOM 20–002, January 2020), and the animals were cared for in accordance with the National Institutes of Health Guide for the Care and Use of Laboratory Animals.

**FIGURE 1:**
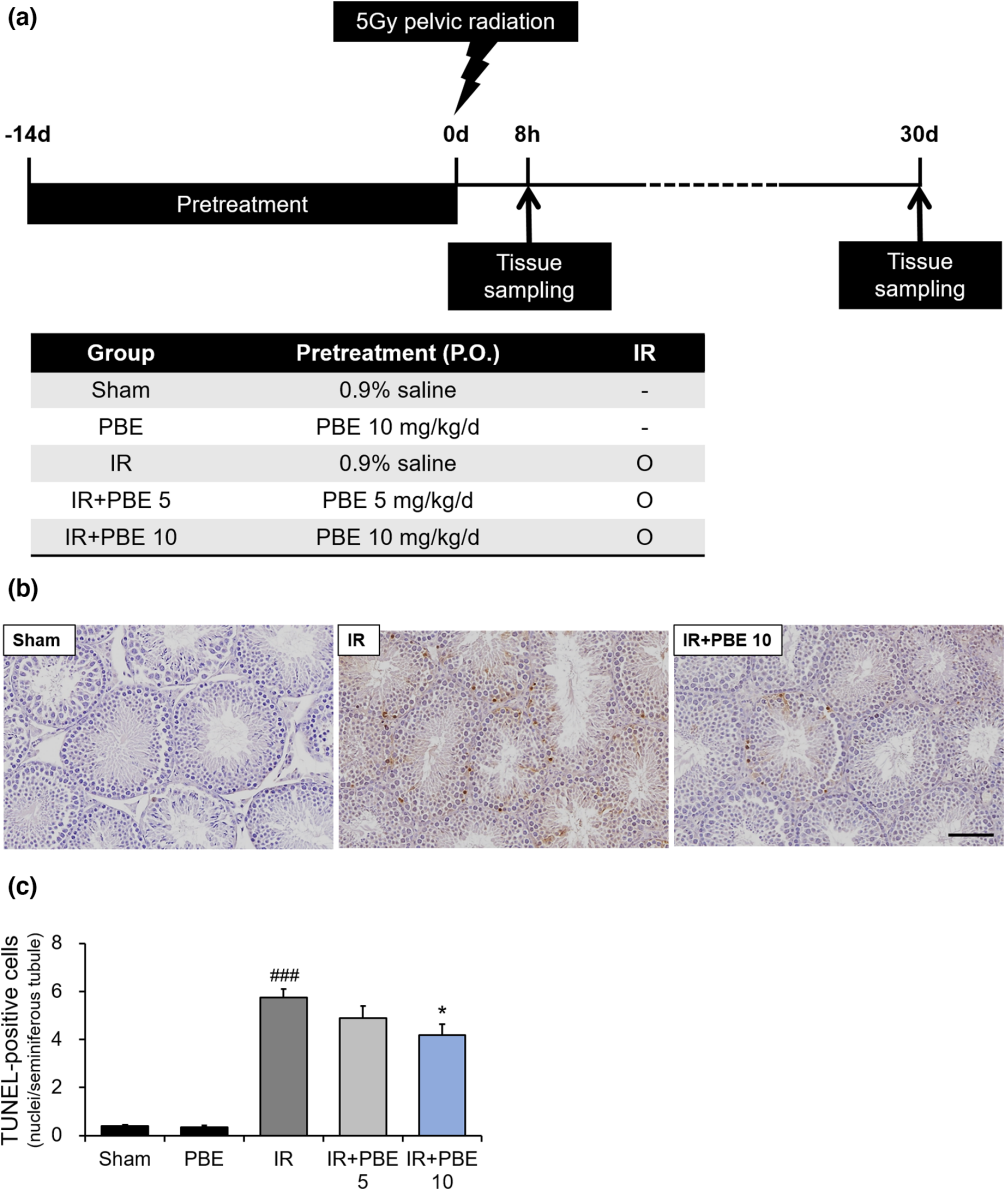
Ameliorative effect of pretreatment with a *Protaetia brevitarsis seulensis* aqueous larval extract against radiation‐induced apoptotic cell death in seminiferous tubules 8 h after pelvic irradiation. (a) Schematic diagram of experimental procedure. (b) Representative photographs (×400) showing TUNEL‐positive apoptotic cells in the testis tissues of mice in the sham, IR, and IR + PBE 10 groups. (c) Graph depicting the mean number of TUNEL‐positive nuclei per seminiferous tubule in each group (*n* = 6 per group). Values are shown as the mean ± SE. ### *p* < 0.001 vs. sham group, * *p* < 0.05 vs. IR group. The scale bar represents 20 μm. IR, pelvic irradiation; PBE, aqueous extract of *P. brevitarsis seulensis* larvae; IR + PBE 10 group, mice pretreated with 10 mg/kg PBE before irradiation; TUNEL, terminal deoxynucleotidyl transferase‐mediated dUTP‐biotin nick end labeling

The animals were randomly divided into the following five groups (*n* = 12 mice per group): 1) sham‐irradiated (sham) group, 2) 10 mg/kg PBE treatment (PBE) group, 3) vehicle‐treated irradiation (IR) group, 4) 5 mg/kg PBE‐treated irradiation (IR + PBE 5) group, and 5) 10 mg/kg PBE‐treated irradiation (IR + PBE 10) group. The experimental procedure is summarized in Figure 1a. Before radiation exposure, the mice in the respective groups were administered PBE (5 or 10 mg/kg) or the vehicle (0.9% saline) daily for 2 weeks by oral gavage. Mice in all the IR groups were subjected to pelvic radiation (5 Gy). The mice were sacrificed at 8 h and 30 days after irradiation (*n* = 6 mice per group), and the testis and epididymis were collected from each mouse.

### 2.3 Radiation exposure

A mouse model of radiation‐induced testicular injury was prepared using a previously described protocol (Lee et al., 2021). Briefly, the mice were anesthetized and their extremities were immobilized on a tray. Using cobalt‐60 gamma rays (Elekta), at a dose rate of 3.8 Gy/min, single fractions of 5 Gy were delivered at a specific depth through a distal pelvic field measuring 2 × 2 cm, which completely encompassed the two testes. Sham‐irradiated mice were treated in exactly the same manner as the irradiated animals but were not exposed to gamma rays.

### 2.4 Apoptosis assay

To ascertain the anti‐apoptotic effect of PBE, several of the mice in each group were sacrificed 8 h after 5 Gy irradiation. The right testis was fixed in Bouin's solution for 2 days and then paraffinized. Sections perpendicular to the long axis of the testis were obtained and stained using the terminal deoxynucleotidyl transferase‐mediated dUTP‐biotin nick end labeling (TUNEL) method using a commercial kit (ApopTag Plus; Intergen). At least 50 tubular cross‐sections were selected randomly from each tissue section, with two sections from each animal examined under a microscope, and the mean number of TUNEL‐positive cells per seminiferous tubule was calculated using a previously described method (Lee et al., 2021).

### 2.5 Histological examination of the testis

To investigate the effects of PBE on the histopathological parameters of the testis and spermatogenesis, the mice were sacrificed 30 days after irradiation, and their right testes were immediately removed and weighed. The testes were fixed in Bouin's solution for 2 days and embedded in paraffin, according to routine methods. Furthermore, sections perpendicular to the long axis of the testis were obtained and stained with hematoxylin and eosin (H&E) solution. The height of the germinal epithelium and the diameter of the seminiferous tubules were measured in the testis sections using an ocular micrometer. At least 30 round tubules (excluding those on the edge of the testis) were measured for each mouse, and their mean diameter and epithelium height were taken as *n* = 1 (Kim et al., 2012). The testicular damage and spermatogenesis were assessed histopathologically using Johnsen's mean testicular biopsy score (MTBS) (Johnsen, 1970). By using H&E stained slides, 30 tubules for each animal were graded and each tubule was given a score from 1 to 10 based on the presence or absence of germ cell types in the testicular seminiferous tubules such as spermatozoa, spermatids, spermatocyte, spermatogonia, germ cells, and Sertoli cells to evaluate histology. A higher Johnsen's score indicates a better status of spermatogenesis, while a lower score refers to more severe dysfunction. Score 1 means no epithelial maturation considered for the tubules with complete inactivity while a score 10 means full epithelial maturation considered for the tubules with maximum activity (Johnsen, 1970).

### 2.6 Evaluation of sperm characteristics

To obtain sperm counts and check for sperm abnormalities, the right caudal epididymis was weighed, placed in saline (0.5 ml), and homogenized for 30 s. A 10 μl aliquot of the sample was then diluted with a solution consisting of 0.25% trypan blue, 5% NaHCO3, and 0.35% formalin, and the sperm cells were counted using a hemocytometer. A portion of the sperm suspension was mixed with 1% eosin Y and then smeared on a glass slide. Four hundred spermatozoa per animal were evaluated for head and tail defects under a light microscope at 400 × magnification. Abnormal sperm morphologies included a small head, an amorphous head, two heads and/or tails, a straight hook, an excessive hook, a folded tail, a short tail, or no tail, as previously described (Kim et al., 2011; Kim et al., 2012).

For the motility assay, the left caudal epididymis was removed, placed in 100 μl of 5 mg/mL bovine serum albumin, and finely minced using a small pair of scissors. After incubation for 10 min at 37 °C, which allowed the sperm to be released into the solution, the cells were collected with a micropipette, and 20 μl of the sample was dispensed onto a slide for motility analysis. Motility was observed using a microscope with a stage warmer. Sperm were considered motile if they showed any movement at all. For each preparation, at least 100 sperm cells were counted in the same field. The number of mobile sperms was determined by counting all motile cells. Sperm motility was assessed using a new preparation from the same semen sample (Kim et al., 2012).

### 2.7 DCFDA assay of the ROS‐scavenging activity of the aqueous larval extract

ROS production in the testicular tissues was quantitated using the DCFDA (2,7‐dichlorodihydrofluorescein diacetate) assay, a method based on the ROS‐dependent oxidation of DCFDA to 2,7‐dichlorofluorescin (DCF). Briefly, after thawing the frozen testicular tissue and homogenizing it in lysis buffer (0.5 ml), the homogenate was centrifuged at 14,000 rpm for 30 min at 4°C, and the pellet was resuspended. DCFDA solution (final concentration 25 μM) was added to the suspension, and the mixture was incubated in the dark for 30 min at 37°C, during which nonfluorescent DCFDA was oxidized to the highly fluorescent DCF in the presence of ROS (LeBel et al., 1992). Changes in fluorescence intensity were measured using a fluorescence plate reader (Paradigm; Beckman Coulter,) at excitation and emission wavelengths of 485 and 530 nm, respectively. The assay was repeated six times for each sample.

### 2.8 Isolation of the components in the aqueous larval extract

The PBE components were isolated using the methods described in our previous study (S. Lee et al., 2021). Briefly, 50.0 g of the aqueous extract was separated on a Diaion HP‐20 gel (Supelco) in a Selekt flash chromatography system (Biotage) using two 340 g Biotage empty cartridges (water:MeOH, 100:0–0:100) to obtain nine fractions (F01 − F09). Of these, F05 (1.55 g) was further fractionated using a flash chromatography system using a 400 g Biotage Sfär C18 D cartridge (water:MeOH, 60:40–0:100), to obtain inosine (122.0 mg) along with 14 subfractions (F0501 − F0514). Furthermore, F0506 (56.1 mg) and F0512 (23.3 mg) were purified using a Waters preparative high performance liquid chromatography (HPLC) system consisting of a 2545Q gradient module and a 2998 photodiode array detector (Waters) equipped with a YMC‐Pack ODS‐A semi‐preparative column (21.2 × 250 mm; 5 μm; 120 Å; water:CH3CN, 98:2–90:10; 6 ml/min; YMC), yielding hypoxanthine (3.4 mg) and uridine (2.9 mg) from F0506, and adenine (2.2 mg) and adenosine (3.1 mg) from F0512. Benzoic acid (7.4 mg) was obtained from F08 (40.1 mg) after purification by preparative HPLC using a YMC‐Pack ODS‐A semi‐preparative column (water:CH3CN, 75:25–60:40; 6 ml/min). Nuclear magnetic resonance (NMR) was performed using a spectraMHz Cryo‐NMR system (Bruker,).

### 2.9 ABTS radical‐scavenging activity of the aqueous larval extract

The ABTS (2,2′‐azinobis[3‐ethylbenzothiazoline‐6‐sulfonic acid]) radical‐scavenging assay was conducted as described in a previous study (Jing et al., 2015) with minor modifications (*n* = 3–4 per group). In brief, the preformed radical monocation of ABTS (ABTS˙+) was first generated by the oxidation of 7 mM ABTS (≥98%; Sigma‐Aldrich,) with 2.5 mM potassium persulfate (≥99%; Sigma‐Aldrich) at a ratio of 1:1. Furthermore, 180 μl of the diluted ABTS + solution (A734 nm = 0.7 ± 0.02) was added to 20 μl each of various concentrations of the PBE (4, 20, 100, 500, and 2500 μg/mL) or isolated compounds (62.5, 125, 250, 500, and 1000 μM), and the mixtures were left to react for 10 min at ambient temperature. Finally, absorbance was measured at 734 nm using a SpectraMax i3x microplate spectrophotometer (Molecular Devices,). The assay was repeated three times for each sample. The percentage inhibition of ABTS radicals was calculated as follows:

$$\text{ABTS inhibition rate}\;\left(\%\right)=\left[1-\left(A_{\text{sample}}-A_{\text{blank}}\right)/\left(A_{\text{control}}-A_{\text{blank}}\right)\right]\times 100.$$

where A represents the absorbance of the indicated (subscripted) items.

### 2.10 Statistical analyses

All data are reported as the mean ± standard error and were analyzed using one‐way analysis of variance, followed by the Student–Newman–Keuls post hoc test for multiple comparisons. In all cases, differences with a *p*‐value of less than 0.05, were considered statistically significant.

## 3 RESULTS

### 3.1 Pretreatment with the larval extract ameliorated radiation‐induced apoptosis in mouse testis

To determine whether PBE pretreatment affects radiation‐induced apoptosis in the seminiferous tubules of the testis, we counted the number of TUNEL‐positive apoptotic nuclei in the testicular germ cells at 8 h after radiation exposure (*n* = 6 mice per group). The representative photographs in Figure 1b show TUNEL‐positive apoptotic cells in the testes of mice from the sham, IR, and IR + PBE 10 groups. The mean number of TUNEL‐positive cells in the seminiferous tubules was 0.41 ± 0.05 and 0.34 ± 0.07 in the sham and PBE groups, respectively. In contrast, the mean number of TUNEL‐positive cells in the IR group was significantly increased by radiation exposure (5.75 ± 0.36; Figure 1c). Although the number of apoptotic nuclei had declined in a dose‐dependent manner in both the IR + PBE 5 (4.90 ± 0.50) and IR + PBE 10 (4.2 ± 0.43) groups, only the latter group showed a statistically significant difference to the IR group in this aspect (Figure 1c).

### 3.2 Pretreatment with the larval extract alleviated weight loss of the testis and epididymis after radiation exposure

**FIGURE 2:**
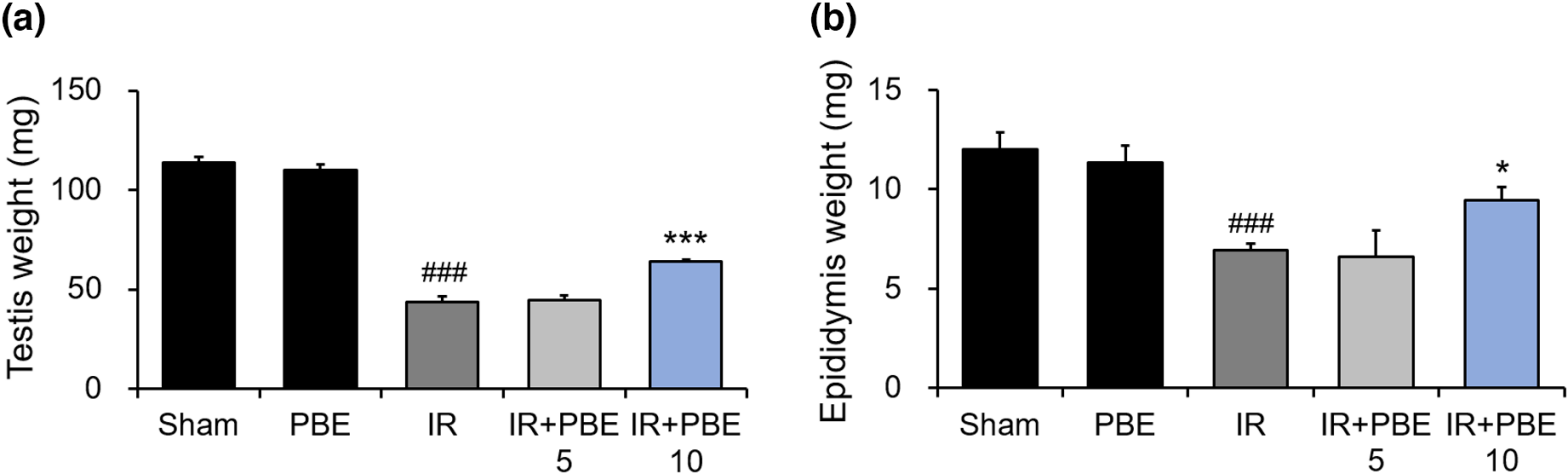
Protective effects of pretreatment with a *Protaetia brevitarsis seulensis* aqueous larval extract against radiation‐induced weight loss of (a) the testis, and (b) epididymis, 30 days after pelvic irradiation (*n* = 6 per group). Values are indicated as the mean ± SE. ### *p* < 0.001 vs. sham group, * *p* < 0.05 and *** *p* < 0.001 vs. IR group. IR, pelvic irradiation; PBE, aqueous extract of *P. brevitarsis seulensis* larvae; IR + PBE 5 group, mice pretreated with 5 mg/kg PBE before irradiation; IR + PBE 10 group, mice pretreated with 10 mg/kg PBE before irradiation

We examined the protective effect of PBE pretreatment against weight loss in the testis and epididymis at 30 days after irradiation (*n* = 6 mice per group). As shown in Figure 2a, the testis weight of mice in the IR group (43.67 ± 2.82 mg) was significantly lower than that of the animals in the sham and PBE groups (113.62 ± 2.85 and 109.85 ± 2.90 mg, respectively). However, the radiation‐induced reduction in testis weight was significantly reduced in the IR + PBE 10 group (64.05 ± 1.09 mg).

Additionally, the epididymis weight of mice in the IR group (6.92 ± 0.33 mg) was also significantly decreased by radiation exposure compared with that of the animals in the sham and PBE groups (12.00 ± 0.86 and 11.33 ± 0.88 mg, respectively; Figure 2b). However, the weight reduction was significantly alleviated by the 10 mg/kg PBE pretreatment before irradiation (9.45 ± 0.68 mg; Figure 2b).

### 3.3 Pretreatment with the larval extract attenuated radiation‐induced damage to the testicular morphology

**FIGURE 3:**
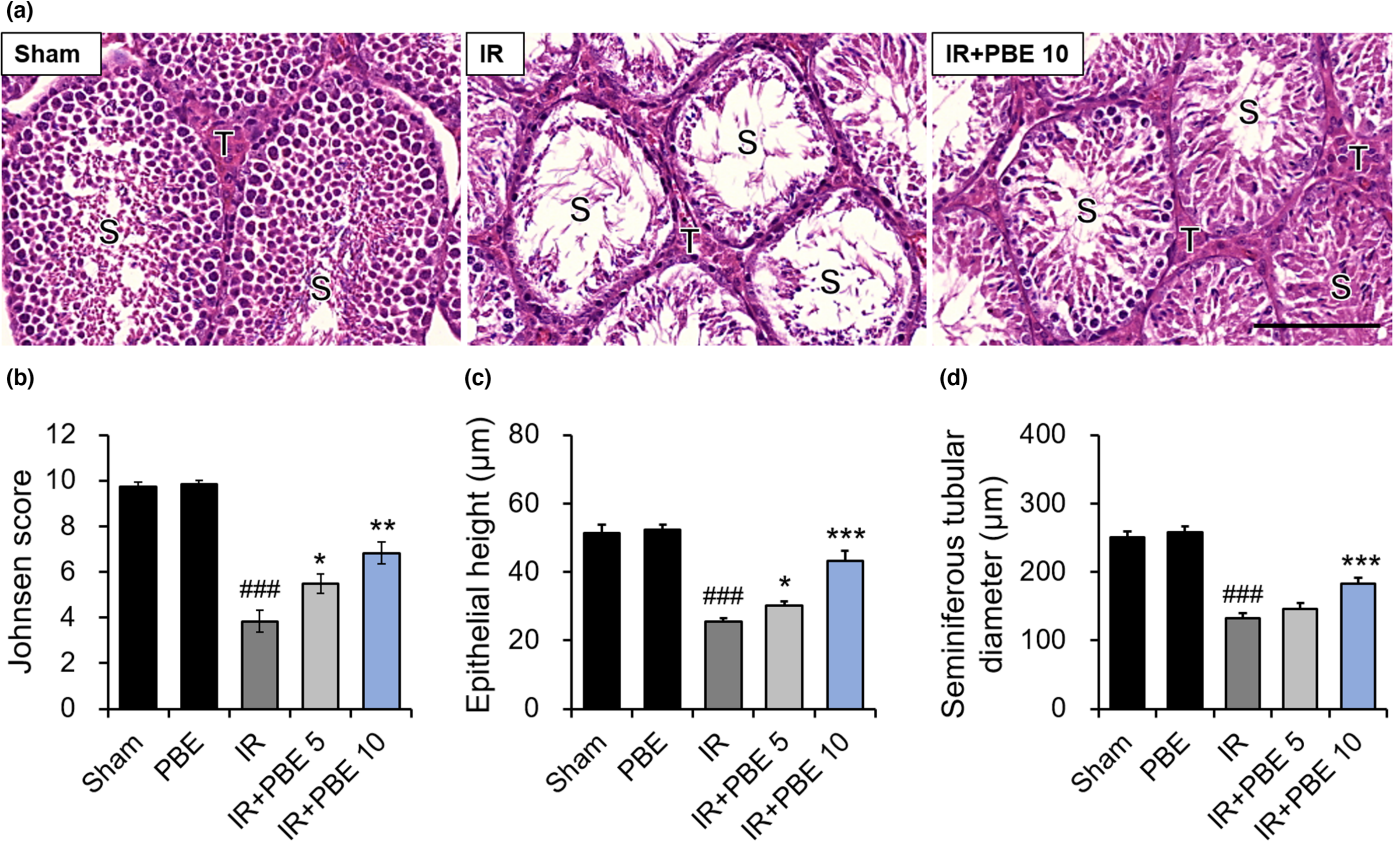
Ameliorative effect of pretreatment with a *Protaetia brevitarsis seulensis* aqueous larval extract on radiation‐induced histopathological changes in the testis 30 days after pelvic irradiation. (a) Representative images (×400) of H&E staining in transverse sections of testicular tissues from mice in the sham, IR, and IR + PBE 10 groups. Quantitative analysis of (b) the epithelium height, and (c) seminiferous tubule diameter (d) in mice of each group (*n* = 6 per group). Values are indicated as the mean ± SE. ### *p* < 0.001 vs. sham group, * *p* < 0.05 and *** *p* < 0.001 vs. IR group. Scale bar represents 20 μm. S, seminiferous tubules; T, interstitial spaces; IR, pelvic irradiation; PBE, aqueous extract of *P. brevitarsis seulensis* larvae; IR + PBE 5 group, mice pretreated with 5 mg/kg PBE before irradiation; IR + PBE 10 group, mice pretreated with 10 mg/kg PBE before irradiation; H&E, hematoxylin and eosin

To investigate the effects of radiation and PBE pretreatment on the morphology of the seminiferous tubules, H&E staining of the testes from the mice in each group was performed 30 days after irradiation. As shown in the representative photographs in Figure 3a, the organs collected from the sham group presented a normal testicular architecture and seminiferous tubular morphology, with primary and secondary spermatocytes, spermatids, and spermatozoa. In contrast, atrophied seminiferous tubules with germ cell loss were observed in the testes of mice in the IR group. In the IR + PBE 10 group, testicular architecture and spermatogenesis were considerably recovered compared with those in the IR group (Figure 3a).

The criteria were formulated by Johnsen to assess murine testicular histology affected by irradiation (Johnsen, 1970). Results showed that Johnsen's score was significantly reduced in the irradiated groups compared with that of the sham group. The seminiferous tubules were graded (on a scale of 1–10) according to the reduction observed in the density of germ cells in the lumen of seminiferous tubules. It was observed that pretreatment with 5 and 10 mg/kg PBE restored the radiation‐induced reductions in the germ cell in the seminiferous tubules and increased the Johnsen scores (Figure 3a,b). To quantitatively evaluate the morphological damage in the testis, we measured the height of the testicular epithelium and the diameter of the seminiferous tubules in each group (*n* = 6 mice per group; Figure 3c,d). The sham and PBE groups showed similar epithelium heights (51.30 ± 2.40 and 52.42 ± 1.45 μm, respectively) and tubule diameters (250.06 ± 9.14 and 258.01 ± 8.70 μm, respectively). In contrast, the epithelium height and tubule diameter were substantially decreased in the IR group (25.51 ± 1.06 and 132.73 ± 6.99 μm, respectively). However, pretreatment with 5 and 10 mg/kg PBE restored the radiation‐induced reductions in the height and diameter values in a dose‐dependent manner (height: 30.15 ± 1.34 and 43.12 ± 3.10 μm, respectively; diameter: 146.53 ± 7.76 and 12.98 ± 8.23 μm, respectively), although the difference in tubule diameter between the IR + PBE 5 and IR groups was not statistically significant.

### 3.4 Pretreatment with the larval extract protected against radiation‐induced changes in the sperm characteristics

**FIGURE 4:**
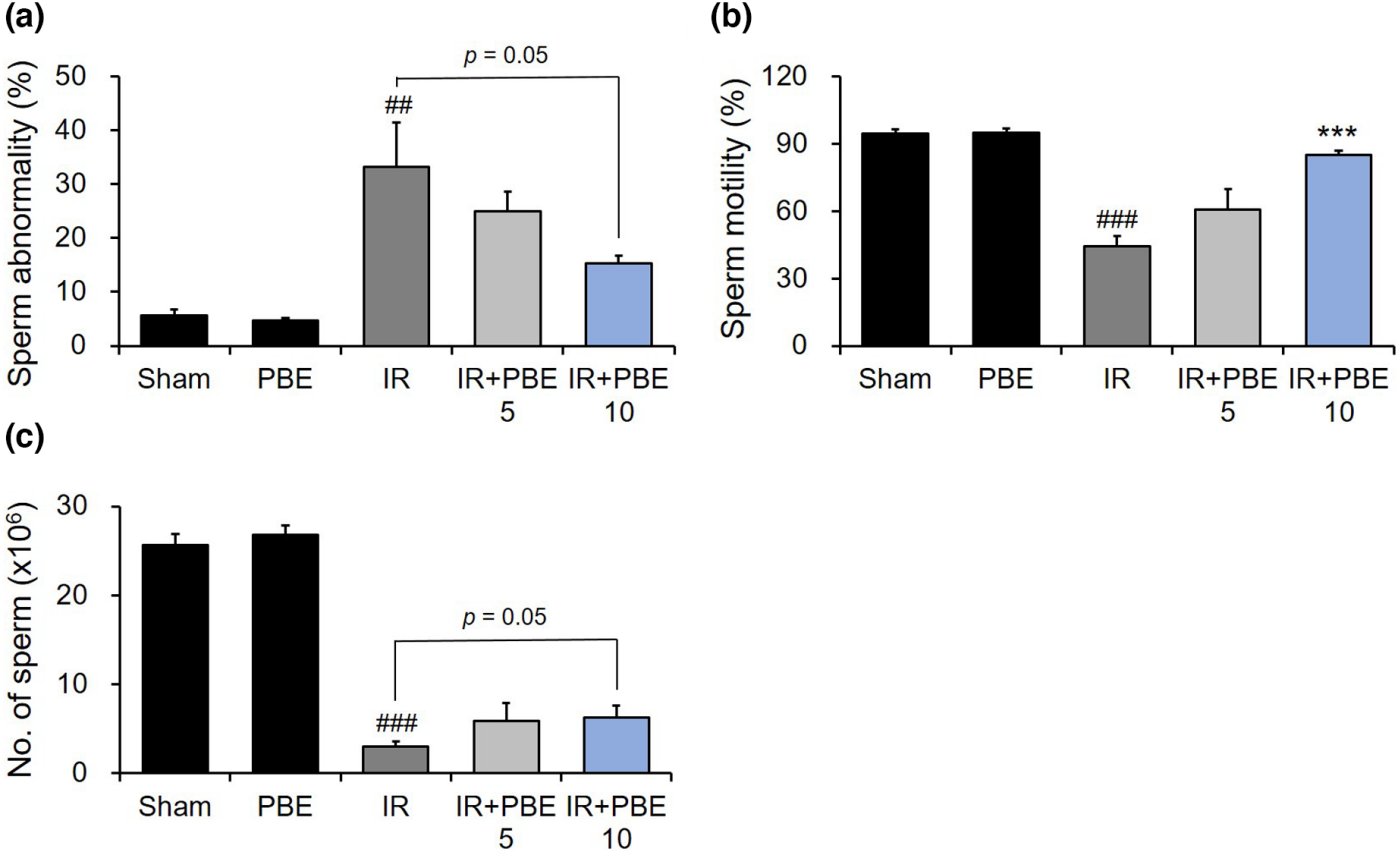
Protective effects of pretreatment with a *Protaetia brevitarsis seulensis* aqueous larval extract against radiation‐induced changes in sperm characteristics 30 days after pelvic irradiation. Quantitative graphs of (a) sperm abnormality, (b) sperm motility, and (c) sperm number in the caudal epididymis of mice in each group (*n* = 6 per group). Values are indicated as the mean ± SE. ## *p* < 0.01 and ### *p* < 0.001 vs. sham group, *** *p* < 0.001 vs. IR group. IR, pelvic irradiation; PBE, aqueous extract of *P. brevitarsis seulensis* larvae; IR + PBE 5 group, mice pretreated with 5 mg/kg PBE before irradiation; IR + PBE 10 group, mice pretreated with 10 mg/kg PBE before irradiation

We confirmed the effects of PBE pretreatment on the characteristics of sperm in the epididymis 30 days after radiation exposure (*n* = 6 mice per group). With regard to the sperm morphology, the sham and PBE groups showed only 5.61 ± 1.19% and 4.67 ± 0.54% of abnormalities, respectively, whereas the IR group exhibited a significantly high percentage (33.20 ± 8.20%) of abnormalities, particularly of sperm cells with detached tails (Figure 4a). With the 10 mg/kg PBE pretreatment, the rate of abnormalities decreased to 15.24 ± 1.54% relative to that of the IR group (*p* = 0.05; Figure 4a).

The percentage sperm motility (i.e., cells with proper movement) was 94.67 ± 1.73% in the sham group and 94.92 ± 1.83% in the PBE group, whereas it was 44.33 ± 4.50% in the IR group, indicating that radiation causes substantial damage to sperm motility (Figure 4b). In contrast, the percentage of mobile cells in the IR + PBE 10 group was 85.00 ± 1.98%, indicating that the 10 mg/kg PBE pretreatment significantly prevented motility injury in the sperms relative to that in the IR group (Figure 4b).

With regard to the sperm count in the caudal epididymis, the number (×106) in the IR group (3.00 ± 0.58) was considerably lower than that in the sham and PBE groups (25.61 ± 1.24 and 26.78 ± 1.09, respectively). However, pretreatment with 10 mg/kg PBE increased the sperm count (6.26 ± 1.36) relative to that in the IR group (*p* = 0.05; Figure 4c).

### 3.5 The aqueous larval extract and its components possessed antioxidative potential

**FIGURE 5:**
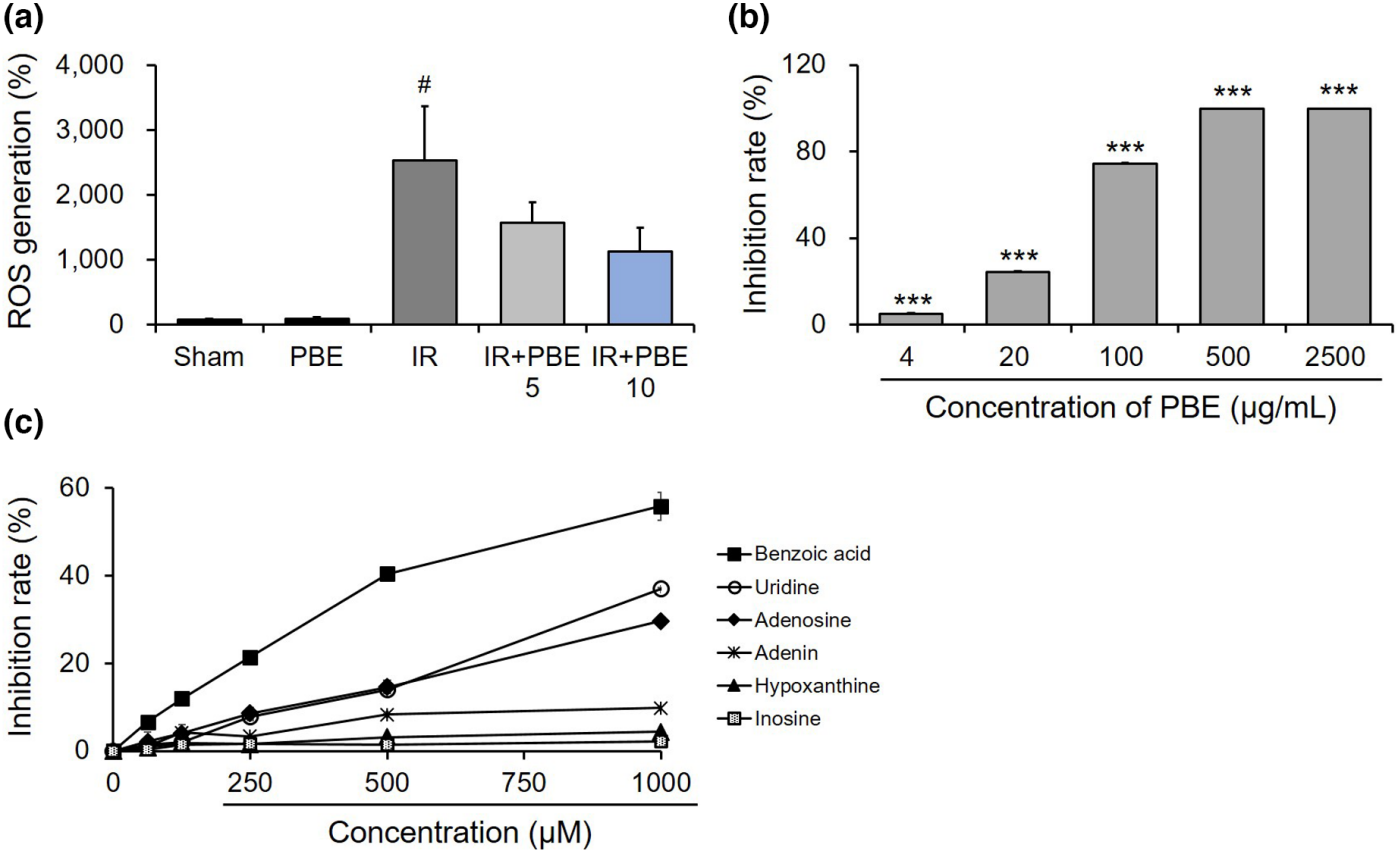
Antioxidative activities of a *Protaetia brevitarsis seulensis* aqueous larval extract in an in vivo model and of the isolated extract components in vitro. (a) Inhibitory effects of 5 and 10 mg/kg PBE pretreatments on radiation‐induced ROS generation in the testis of mice in each group at 30 days after pelvic irradiation (*n* = 6 per group). (b,c) ABTS assays of the free radical‐scavenging activities of the PBE (b) and six isolated compounds (c) (*n* = 3–4 per group). Values are shown as the mean ± SE. # *p* < 0.05 vs. sham group, *** *p* < 0.001 vs. non‐PBE‐treated group. IR, pelvic irradiation; PBE, aqueous extract of *P. brevitarsis seulensis* larvae; IR + PBE 5 group, mice pretreated with 5 mg/kg PBE before irradiation; IR + PBE 10 group, mice pretreated with 10 mg/kg PBE before irradiation; ROS, reactive oxygen species

To evaluate whether PBE pretreatment reduces ROS generation to protect against radiation‐induced oxidative stress in the testis, we measured the levels of DCFDA in the tissue samples 30 days after irradiation (*n* = 6 mice per group; Figure 5a). The ROS levels in the sham and PBE groups were 74.00 ± 10.16% and 93.33 ± 25.25%, respectively, whereas the level in the IR group considerably increased after radiation exposure to 2532.00 ± 837.33%. Although the 5 and 10 mg/kg PBE pretreatment prevented ROS generation in the testis in a dose‐dependent manner (1573.00 ± 313.30% and 1125.67 ± 373.11%, respectively), the differences in ROS levels in the IR group were not statistically significant.

Furthermore, the ABTS radical‐scavenging assay was performed to confirm the antioxidative capacity of PBE and to determine which of its components act as antioxidants. The inhibition rate achieved by using PBE was significantly higher than that of the blank control, with inhibition occurring in a dose‐dependent manner above the PBE concentration of 4 μg/mL (*n* = 4 per group; Figure 5b). The inhibition rate appeared to reach a plateau above the PBE concentration of 500 μg/mL, and the half inhibitory concentration (IC50) was 43.36 μg/mL. Among the components isolated from the extract, benzoic acid, uridine, and adenosine displayed significantly higher inhibition rates in a dose‐dependent manner at concentrations above 62.5, 125, and 250 μM, respectively, compared with the rates observed in the blank control group (*n* = 3 per group; Figure 5c). The IC50 values of benzoic acid, uridine, and adenosine against the ABTS radical were 806.77, 1821.50, and 1695.32 μM, respectively.

## 4 DISCUSSION

Radiotherapy, which is widely used to treat various types of cancer and to enhance patient survival, can cause side effects in long‐term cancer survivors. Although radiation can effectively decrease cancer cells, it can also damage healthy tissues. The testis is one of the most radiosensitive organs, and its exposure to radiation can result in tissue damage, structural and cellular changes, and functional impairment of spermatogenesis, such as dysfunction in sperm metabolism, differentiation, and proliferation, resulting in a reduction in the number of spermatozoa and damage to the spermatids.

Studies have shown that polydatin exerts protective effects against radiation‐induced structural damage to the testes and loss of spermatophores (S. Khan et al., 2015; Ma & Jia, 2018). Therefore, we investigated the radioprotective effects of an extract from another abundant natural resource, *P. brevitarsis seulensis* larvae, on irradiated mouse testes. Increased expression of apoptotic nuclei in the germ cell was observed in the damaged testis of irradiated mice, and these morphologic characteristics can be confirmed through TUNEL‐positive cell staining (Haeri et al., 2014; W. Lee et al., 2015). Increase in apoptotic nuclei was observed in the damaged seminiferous tubules, but it was observed that the TUNEL‐positive cells in the groups that had undergone pretreatment with PBE was reduced. H&E staining was also used to determine whether the structural changes to the organ and the loss of spermatophores could be alleviated by PBE pretreatment. We found that PBE pretreatment restored the height of the epithelium and the diameter of the seminiferous tubules, which had reduced due to radiation. Furthermore, PBE ameliorated the deleterious changes induced in the seminiferous tubule morphology, spermatogenesis, and sperm mobility, but had no effect on the number of sperm and spermatids. In other papers that confirm the effect of natural resources or derived ingredients, protective effects against damaged seminiferous tubules have been demonstrated through the degree to which the height of the epithelium and the diameter of the seminiferous tubules and sperm mobility were restored (Bin‐Meferij & El‐Kott, 2015; Haeri et al., 2014).

Oxidative stress is caused by the generation of ROS, which results in tissue damage by inducing molecular changes, damaging DNA, lipids, and proteins, and altering signal transduction pathways in germ cells (Aitken & Roman, 2008). Research studies have shown that radiation‐induced oxidative stress mediates the production of free radicals, resulting in testicular somatic or germ cell death via the apoptotic pathway. In addition to its association with cell death and cellular senescence, ROS generation is also implicated in severe oxidative damage of the testes, such as reduction in the seminiferous tubule diameter, sperm count, and sperm viability (Ameli et al., 2019; Shahin et al., 2018). The DEFCA assay was used in this study, the ROS levels in the IR group significantly increased, whereas the PBE pretreatment had a tendency to suppress the ROS generation in the testis. Despite the difference was insignificant, the ABTS assay revealed that PBE has a substantial ability to prevent ROS generation. As a conclusion, we could establish that the PBE pretreatment suppresses ROS production, protecting it against radiation‐induced oxidative damage in the testis.

Several preclinical studies have revealed that therapies aimed at reducing oxidative stress are effective in modulating radiation‐induced deleterious effects. For example, pretreatment of cells with manganese superoxide dismutase–plasmid liposomes proved effective in protecting them against radiation‐induced damage. In this study, we have demonstrated using the ABTS assay that PBE has free radical‐scavenging potential, similar to that of nitric oxide, verifying the ROS removal capacity of the extract, as reported by other studies (Lee et al., 2019; Lee et al., 2021). Although larvae of *P. brevitarsis seulensis* have a long history of use in traditional East Asian medicine for the treatment of several diseases, no studies have been conducted on its protective effect against testicular diseases. In our previous studies, we confirmed that the main components of PBE were inosine, benzoic acid, adenine, adenosine, hypoxanthine, and uridine (Lee et al., 2021). Among these, uridine, inosine, and benzoic acid are known to display antioxidative activity (Augusto et al., 2014; Bergman et al., 2001; Gudkov et al., 2006; Lee et al., 2021); these data were reconfirmed in the current study, particularly for benzoic acid and uridine.

We also found that abdominal irradiation induced germ cell apoptosis, decreased the testis and epididymis weights, suppressed the epithelium height and seminiferous tubule diameter, and reduced the number of sperm and spermatids and their mobility, all of which were revealed 30 days after radiation exposure (Haeri et al., 2014; Ji et al., 2016; Shahin et al., 2018). Importantly, our results showed that pre‐administration of PBE to mice inhibited these detrimental testicular injuries and improved sperm quantity and quality. Moreover, pretreatment with PBE significantly increased the diameter of seminiferous tubules and recovered the size of the testicles relative to that in the IR group. Although the medical effect of PBE could be confirmed through this study, further research to establish optimal extraction conditions for maximizing the efficacy of the extract should be conducted, and additional chemical analysis studies are needed to identify the composition including useful proteins.

To the best of our knowledge, this study is the first to demonstrate the protective effects of PBE and its individual components against radiation‐induced testicular damage and spermatogenesis impairment. Although further studies on the correlation between these components and their radioprotective effects and their underlying mechanisms are needed, the results of this study highlight the potential clinical applicability of PBE as a neoadjuvant therapy for the prevention of testicular damage caused by cancer radiotherapy.

In conclusion, our study demonstrates that pelvic irradiation can cause harm to the testes through the generation of ROS. Furthermore, we revealed that PBE scavenges ROS, thereby protecting the organs from radiation‐induced injury. Therefore, PBE has high potential for use in clinical practice to provide protection against testicular injury from radiotherapy.

## AUTHOR CONTRIBUTIONS

Hyeon‐Hwa Nam: Formal analysis (lead); Investigation (equal); Writing–original draft (equal); Writing–review and editing (equal). Sohi Kang: Data curation (equal); Formal analysis (equal); Investigation (lead). Yun‐Soo Seo: Formal analysis (equal); Investigation (equal). Jun Lee and Byeong Cheol Moon: Data curation (equal); Formal analysis (equal). Hae June Lee: Methodology (Radiation exposure). Ji Hye Lee: Data curation (equal). Bohae Kim: Investigation (Histopathological exam). Sueun Lee: Data curation (equal); Formal analysis (equal); Investigation (equal); Writing–original draft (lead); Writing–review and editing (equal). Joong‐Sun Kim: Conceptualization (lead); Data curation (equal); Methodology (equal); Writing–original draft (lead); Writing–review and editing (equal).

## CONFLICT OF INTEREST

The authors declare that they do not have any conflicts of interest.

## ETHICAL APPROVAL

All animal experiments followed a protocol approved by the Institutional Animal Care and Use Committee of the Korea Institute of Oriental Medicine (KIOM 20–002).

## INFORMED CONSENT

Written informed consent was obtained from all study participants.

## References

[fsn32992-bib-0001] Aitken, R. J. , & Roman, S. D. (2008). Antioxidant systems and oxidative stress in the testes. Oxidative Medicine and Cellular Longevity, 1(1), 154–171. 10.4161/oxim.1.1.6843 PMC2715191 19794904

[fsn32992-bib-0002] Ameli, M. , Moghimian, M. , Saeb, F. , Bashtani, E. , Shokoohi, M. , Salimnejad, R. , & Abtahi, H. (2019). The effect of clomiphene citrate and human chorionic gonadotropin on the expression of CatSper1, CatSper2, LHCGR, and SF1 genes, as well as the structural changes in testicular tissue of adult rats. Molecular Reproduction and Development, 86(6), 738–748. 31041823 10.1002/mrd.23151

[fsn32992-bib-0003] Andreu, J. A. L. , Fernandez, P. J. , Tortajada, J. F. i. , Navarro, I. , Rodriguez‐Ineba, A. , Muro, M. D. , & Romeu, A. (2000). Persistent altered spermatogenesis in long‐term childhood cancer survivors. Pediatric Hematology and Oncology, 17(1), 21–30. 10689712 10.1080/088800100276631

[fsn32992-bib-0004] Augusto, T. , Salinas, E. , Alencar, S. , & D'arce, M. (2014). Camargo ACd, Vieira TMFdS phenolic compounds and antioxidant activity of hydroalcoholic extracts of wild and cultivated murtilla (Ugni molinae Turcz) Food Sci. Technology, 34, 667–679.

[fsn32992-bib-0005] Baek, S. , Noh, H. H. , Kim, C. J. , Son, K. , Lee, H.‐D. , & Kim, L. (2021). Easy and effective analytical method of carbendazim, dimethomorph, and fenoxanil from Protaetia brevitarsis seulensis using LC‐MS/MS. PLoS One, 16(10), e0258266. 34648540 10.1371/journal.pone.0258266 PMC8516223

[fsn32992-bib-0006] Bergman, M. , Varshavsky, L. , Gottlieb, H. E. , & Grossman, S. (2001). The antioxidant activity of aqueous spinach extract: Chemical identification of active fractions. Phytochemistry, 58(1), 143–152. 10.1016/s0031-9422(01)00137-6 11524124

[fsn32992-bib-0007] Bin‐Meferij, M. M. , & El‐Kott, A. F. (2015). The radioprotective effects of Moringa oleifera against mobile phone electromagnetic radiation‐induced infertility in rats. International Journal of Clinical and Experimental Medicine, 8(8), 12487–12497. 26550159 PMC4612844

[fsn32992-bib-0008] Chatterjee, J. , Nairy, R. K. , Langhnoja, J. , Tripathi, A. , Patil, R. K. , Pillai, P. P. , & Mustak, M. S. (2018). ER stress and genomic instability induced by gamma radiation in mice primary cultured glial cells. Metabolic Brain Disease, 33(3), 855–868. 29429012 10.1007/s11011-018-0183-9

[fsn32992-bib-0009] Feng, Y. , Zhao, M. , He, Z. , Chen, Z. , & Sun, L. (2009). Research and utilization of medicinal insects in China. Entomological Research, 39(5), 313–316.

[fsn32992-bib-0010] Ganguly, K. , Jeong, M. S. , Dutta, S. D. , Patel, D. K. , Cho, S. J. , & Lim, K. T. (2020). Protaetia brevitarsis seulensis derived protein isolate with enhanced osteomodulatory and antioxidative property. Molecules, 25(24), 6056. 10.3390/molecules25246056 PMC7767527 33371481

[fsn32992-bib-0011] Gudkov, S. V. , Shtarkman, I. N. , Smirnova, V. S. , Chernikov, A. V. , & Bruskov, V. I. (2006). Guanosine and inosine display antioxidant activity, protect DNA in vitro from oxidative damage induced by reactive oxygen species, and serve as radioprotectors in mice. Radiation Research, 165(5), 538–545. 10.1667/rr3552.1 16669708

[fsn32992-bib-0012] Haeri, S. , Rajabi, H. , Fazelipour, S. , & Hosseinimehr, S. (2014). Carnosine mitigates apoptosis and protects testicular seminiferous tubules from gamma‐radiation‐induced injury in mice. Andrologia, 46(9), 1041–1046. 24215656 10.1111/and.12193

[fsn32992-bib-0013] Hofman, M. , Morrow, G. R. , Roscoe, J. A. , Hickok, J. T. , Mustian, K. M. , Moore, D. F. , Wade, J. L. , & Fitch, T. R. (2004). Cancer patients' expectations of experiencing treatment‐related side effects: a university of rochester cancer center‐community clinical oncology program study of 938 patients from community practices. Cancer, 101(4), 851–857. 15305419 10.1002/cncr.20423

[fsn32992-bib-0014] Ji, H. J. , Wang, D. M. , Wu, Y. P. , Niu, Y. Y. , Jia, L. L. , Liu, B. W. , Feng, Q. J. , & Feng, M. L. (2016). Wuzi Yanzong pill, a Chinese polyherbal formula, alleviates testicular damage in mice induced by ionizing radiation. BMC Complementary and Alternative Medicine, 16(1), 509. 10.1186/s12906-016-1481-6 27927244 PMC5142375

[fsn32992-bib-0015] Jing, L. , Ma, H. , Fan, P. , Gao, R. , & Jia, Z. (2015). Antioxidant potential, total phenolic and total flavonoid contents of rhododendron anthopogonoides and its protective effect on hypoxia‐induced injury in PC12 cells. BMC Complementary and Alternative Medicine, 15(1), 1–12. 26283543 10.1186/s12906-015-0820-3 PMC4539926

[fsn32992-bib-0016] Johnsen, S. G. (1970). Testicular biopsy score count–a method for registration of spermatogenesis in human testes: Normal values and results in 335 hypogonadal males. Hormone Research in Pædiatrics, 1(1), 2–25. 10.1159/000178170 5527187

[fsn32992-bib-0017] Khan, H. A. , & Alhomida, A. S. (2011). A review of the logistic role of L‐carnitine in the management of radiation toxicity and radiotherapy side effects. Journal of Applied Toxicology, 31(8), 707–713. 10.1002/jat.1716 21818761

[fsn32992-bib-0018] Khan, S. , Adhikari, J. S. , Rizvi, M. A. , & Chaudhury, N. K. (2015). Radioprotective potential of melatonin against 60Co γ‐ray‐induced testicular injury in male C57BL/6 mice. Journal of Biomedical Science, 22(1), 1–15. 26205951 10.1186/s12929-015-0156-9 PMC4514449

[fsn32992-bib-0019] Kim, J. , Lee, S. , Jeon, B. , Jang, W. , Moon, C. , & Kim, S. (2011). Protection of spermatogenesis against gamma ray‐induced damage by granulocyte colony‐stimulating factor in mice. Andrologia, 43(2), 87–93. 21382061 10.1111/j.1439-0272.2009.01023.x

[fsn32992-bib-0020] Kim, J. S. , Heo, K. , Yi, J. M. , Gong, E. J. , Yang, K. , Moon, C. , & Kim, S. H. (2012). Genistein mitigates radiation‐induced testicular injury. Phytotherapy Research, 26(8), 1119–1125. 22162311 10.1002/ptr.3689

[fsn32992-bib-0021] Kim, W. , Lee, S. , Seo, D. , Kim, D. , Kim, K. , Kim, E. , Kang, J. , Seong, K. M. , Youn, H. , & Youn, B. (2019). Cellular stress responses in radiotherapy. Cells, 8(9), 1105. 10.3390/cells8091105 PMC6769573 31540530

[fsn32992-bib-0022] LeBel, C. P. , Ischiropoulos, H. , & Bondy, S. C. (1992). Evaluation of the probe 2′, 7′‐dichlorofluorescin as an indicator of reactive oxygen species formation and oxidative stress. Chemical Research in Toxicology, 5(2), 227–231. 1322737 10.1021/tx00026a012

[fsn32992-bib-0023] Lee, H. J. , Seo, M. , Lee, J. H. , Kim, I.‐W. , Kim, S. Y. , Hwang, J.‐S. , & Kim, M. (2019). Inhibitory effect of Protaetia brevitarsis seulensis ethanol extract on neuroinflammation in LPS‐stimulated BV‐2 microglia. Journal of Life Science, 29(10), 1096–1103.

[fsn32992-bib-0024] Lee, J. , Hwang, I. H. , Kim, J. H. , Kim, M. , Hwang, J. S. , Kim, Y. H. , & Na, M. (2017). Quinoxaline‐, dopamine‐, and amino acid‐derived metabolites from the edible insect Protaetia brevitarsis seulensis. Archives of Pharmacal Research, 40(9), 1064–1070. 28780757 10.1007/s12272-017-0942-x

[fsn32992-bib-0025] Lee, S. , Seo, Y. H. , Song, J. H. , Kim, W. J. , Lee, J. H. , Moon, B. C. , Ang, M. J. , Kim, S. H. , Moon, C. , & Lee, J. (2021). Neuroprotective effect of Protaetia brevitarsis seulensis' water extract on Trimethyltin‐induced seizures and hippocampal neurodegeneration. International Journal of Molecular Sciences, 22(2), 679. 10.3390/ijms22020679 PMC7827571 33445535

[fsn32992-bib-0026] Lee, W. , Son, Y. , Jang, H. , Bae, M. J. , Kim, J. , Kang, D. , & Kim, J. S. (2015). Protective effect of administered rolipram against radiation‐induced testicular injury in mice. The World Journal of Men's Health, 33(1), 20–29. 10.5534/wjmh.2015.33.1.20 PMC4412004 25927059

[fsn32992-bib-0027] Ma, Y. , & Jia, X. (2018). Polydatin alleviates radiation‐induced testes injury by scavenging ROS and inhibiting apoptosis pathways. Medical science monitor: International medical journal of experimental and clinical research, 24, 8993–9000. 30539937 10.12659/MSM.913725 PMC6299782

[fsn32992-bib-0028] MFDS . (2021). Expand recognition of raw materials for edible insect food. Retrieved from http://www.foodsafetykorea.go.kr/foodcode/01_03.jsp?idx=816

[fsn32992-bib-0029] Moghimian, M. , Soltani, M. , Abtahi, H. , & Shokoohi, M. (2017). Effect of vitamin C on tissue damage and oxidative stress following tunica vaginalis flap coverage after testicular torsion. Journal of Pediatric Surgery, 52(10), 1651–1655. 28760456 10.1016/j.jpedsurg.2017.07.001

[fsn32992-bib-0030] Okada, K. , & Fujisawa, M. (2019). Recovery of spermatogenesis following cancer treatment with cytotoxic chemotherapy and radiotherapy. The world journal of men's health, 37(2), 166–174. 10.5534/wjmh.180043 PMC6479085 30588779

[fsn32992-bib-0031] Panganiban, R.‐A. M. , Snow, A. L. , & Day, R. M. (2013). Mechanisms of radiation toxicity in transformed and non‐transformed cells. International Journal of Molecular Sciences, 14(8), 15931–15958. 23912235 10.3390/ijms140815931 PMC3759894

[fsn32992-bib-0032] Rozati, H. , Handley, T. , & Jayasena, C. (2017). Process and pitfalls of sperm cryopreservation. Journal of Clinical Medicine, 6(9), 89. 10.3390/jcm6090089 PMC5615282 28925939

[fsn32992-bib-0033] Shahin, S. , Singh, S. P. , & Chaturvedi, C. M. (2018). 2.45 GHz microwave radiation induced oxidative and nitrosative stress mediated testicular apoptosis: Involvement of a p53 dependent bax‐caspase‐3 mediated pathway. Environmental Toxicology, 33(9), 931–945. 29968967 10.1002/tox.22578

[fsn32992-bib-0034] Shokoohi, M. , Khaki, A. , Abadi A., R. R. , MohammadZadeh Boukani, L. , Hassanpour Khodaie, S. , Kalarestaghi, H. , Khaki, A. A. , Moghimian, M. , Niazkar, H. R. , & Shoorei, H. (2022). Minocycline can reduce testicular apoptosis related to varicocele in male rats. Andrologia, 54(4), e14375. 35266181 10.1111/and.14375

[fsn32992-bib-0035] Shokoohi, M. , Khaki, A. , Shoorei, H. , Khaki, A. , Moghimian, M. , & Abtahi‐Eivary, S. H. (2020). Hesperidin attenuated apoptotic‐related genes in testicle of a male rat model of varicocoele. Andrology, 8(1), 249–258. 31325243 10.1111/andr.12681

[fsn32992-bib-0036] Soltani, M. , Moghimian, M. , Abtahi‐Eivari, S. H. , Shoorei, H. , Khaki, A. , & Shokoohi, M. (2018). Protective effects of matricaria chamomilla extract on torsion/detorsion‐induced tissue damage and oxidative stress in adult rat testis. International journal of fertility & sterility, 12(3), 242–248. 29935071 10.22074/ijfs.2018.5324 PMC6018175

[fsn32992-bib-0037] Srinivas, U. S. , Tan, B. W. Q. , Vellayappan, B. A. , & Jeyasekharan, A. D. (2019). ROS and the DNA damage response in cancer. Redox Biology, 25, 101084. 10.1016/j.redox.2018.101084 30612957 PMC6859528

[fsn32992-bib-0038] Trost, L. W. , & Brannigan, R. E. (2012). Oncofertility and the male cancer patient. Current Treatment Options in Oncology, 13(2), 146–160. 22528369 10.1007/s11864-012-0191-7

[fsn32992-bib-0039] Yeo, H. , Youn, K. , Kim, M. , Yun, E.‐Y. , Hwang, J. S. , Jeong, W. S. , & Jun, M. (2013). Fatty acid composition and volatile constituents of Protaetia brevitarsis larvae. Preventive Nutrition and Food Science, 18(2), 150–156. 24471125 10.3746/pnf.2013.18.2.150 PMC3892504

